# Beyond Neutropenic Fever: Severe Multiorgan Immune-Related Toxicity Induced by Pembrolizumab

**DOI:** 10.7759/cureus.100567

**Published:** 2026-01-01

**Authors:** Marta Anastácio, Tiago Pina Cabral, Marta Roldão, Andreia Salgadinho Machado, Ana Lynce

**Affiliations:** 1 Internal Medicine, Centro Hospitalar de Lisboa Ocidental, Lisbon, PRT; 2 Medical Oncology, Hospital de São Francisco Xavier, Lisbon, PRT; 3 Internal Medicine, Hospital de São Francisco Xavier, Lisbon, PRT

**Keywords:** adrenal insufficiency (ai), immune-checkpoint inhibitors, multiorgan toxicity, oncologic emergency, pembrolizumab toxicity

## Abstract

The use of immune checkpoint inhibitors (ICIs) has revolutionized cancer therapy, introducing a new spectrum of oncologic emergencies associated with immune-related toxicities, which are sometimes severe and difficult to recognize. We report the case of a 49-year-old woman with early-stage triple-negative breast cancer undergoing neoadjuvant chemotherapy with carboplatin, paclitaxel, and pembrolizumab, who presented to the emergency department with abdominal pain, diarrhea, nausea, and vomiting. On admission, she was in shock, with pancytopenia, acute kidney injury, hyponatremia, and low serum cortisol levels. After exclusion of infectious causes, she was diagnosed with pembrolizumab-induced multiorgan immune-related toxicity, manifesting as acute adrenal insufficiency with shock (grade 3), colitis (grade 3), pancytopenia (grade 3), and hypothyroidism (grade 2). Treatment with methylprednisolone 500 mg/day for three days, followed by tapering, filgrastim, and levothyroxine adjustment, led to rapid clinical and laboratory improvement. Pembrolizumab was permanently discontinued. This case highlights the importance of early recognition and aggressive management of potentially fatal immune-related toxicities associated with ICIs.

## Introduction

Immune checkpoint inhibitors (ICIs), such as pembrolizumab, have transformed the oncologic therapeutic landscape by promoting durable antitumor responses through the reactivation of antigen-specific immunity [1-3]. However, this immune activation can disrupt self-tolerance and trigger immune-related adverse events (irAEs) that affect multiple organs and systems [1,2,4,5]. These toxicities differ from the classic adverse reactions associated with chemotherapy, exhibiting substantial clinical heterogeneity and often requiring a rigorous, exclusion-based differential diagnosis [2-4,6]. The most common manifestations are cutaneous, gastrointestinal, pulmonary, endocrine, and hematologic events, whereas severe multiorgan toxicities remain rare but potentially fatal [2,3,6,7].

Adrenal insufficiency induced by ICIs is an uncommon complication, most frequently associated with hypophysitis and secondary adrenocorticotropic hormone (ACTH) deficiency, which contributes to delayed recognition [6,8-11]. Cases combining adrenal insufficiency, colitis, pancytopenia, and thyroid dysfunction are rare, underscoring the importance of individual case reports in expanding the clinical understanding of the irAEs spectrum [4,6,7,9,12].

We report a unique case of pembrolizumab-induced multiorgan immune-related toxicity manifested by adrenal crisis with shock (grade 4), colitis (grade 3), pancytopenia (grade 3), and thyroiditis evolving to clinically significant hypothyroidism (grade 2), with favorable evolution following high-dose corticosteroid therapy. This case reinforces the importance of early recognition, multidisciplinary management, and prompt immunosuppressive treatment to prevent severe morbidity and mortality associated with irAEs.

## Case presentation

A 49-year-old woman, independent in activities of daily living, with a personal medical history of early-stage right-sided triple-negative breast cancer (stage IA), is receiving neoadjuvant chemotherapy with carboplatin, paclitaxel, and pembrolizumab (third cycle), and Hashimoto’s thyroiditis on levothyroxine 25 µg/day. She had no recent travel history.

She presented to the emergency department with abdominal pain localized to the lower quadrants (7/10 intensity), nonradiating, without relieving or aggravating factors, associated with nausea, vomiting, and profuse watery diarrhea (more than 10 episodes in 12 hours), without macroscopic blood. On examination, she was febrile (38.2°C), hypotensive (87/55 mmHg), tachycardic (127 bpm), and hypoglycemic (55 mg/dL), with dehydrated mucosae and diffuse abdominal tenderness on deep palpation.

Initial laboratory evaluation revealed pancytopenia, Acute Kidney Injury Network (AKIN) II, hyponatremia, and elevated C-reactive protein and procalcitonin. Thyroid function testing showed significant dysfunction. The arterial lactate levels were within the normal range. The laboratory results are presented in Table 1 below.

**Table 1:** Laboratory results at hospital admission TSH: thyroid-stimulating hormone

Test	Result	Normal range
Hemoglobin (g/dL)	8.1	12.0-13.0
Leukocytes (x10⁹/L)	0.8	4.0-10.0
Neutrophils (x10⁹/L)	0.7	1.5-8.0
Platelets (x10⁹/L)	45	150-400
Creatinine (mg/dL)	1.78	0.5-0.9
Sodium (mmol/L)	128	136-145
C-reactive protein (mg/dL)	18.3	<0.5
Procalcitonin (ng/mL)	0.19	<0.1
TSH (μIU/mL)	72	0.4-4.5
Free T4 (ng/dL)	2.28	0.7-1.8
Arterial lactate (mmol/L)	2.1	0.5-2.2

An initial diagnosis of febrile neutropenia with suspected abdominal infection was made. A full infectious workup was performed, and empiric antibiotic therapy with piperacillin-tazobactam 4.5 g every six hours was initiated, along with fluid resuscitation (30 mL/kg of normal saline). Despite aggressive volume replacement, the patient remained hypotensive and progressed to shock within hours of presentation, prompting initiation of norepinephrine infusion.

**Figure 1:**
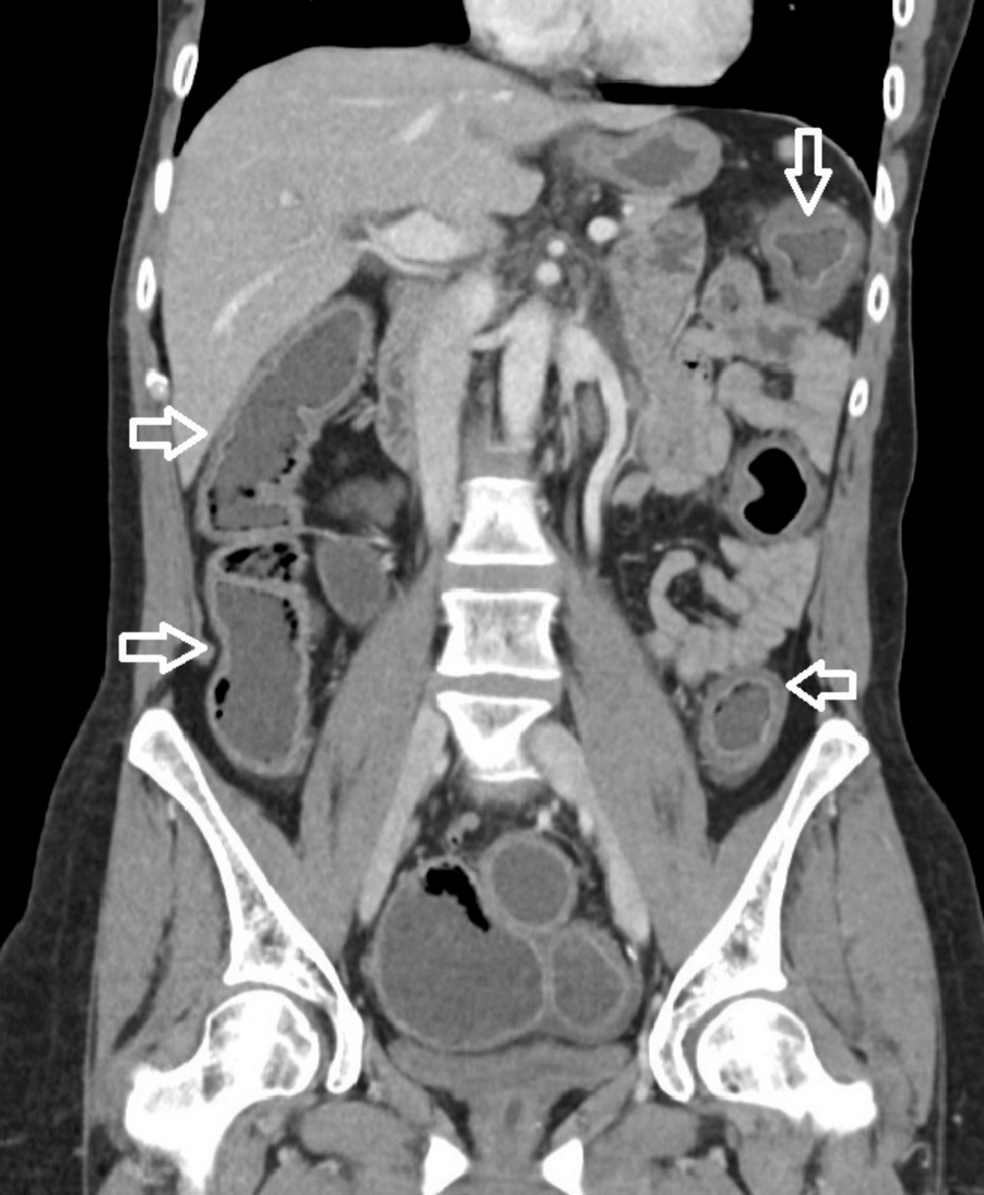
Coronal view of abdominopelvic CT Scan with contrast-the four white arrows point to diffuse colonic wall thickening and enhancement related to pancolitis, without evidence of bowel perforation or obstruction CT: computed tomography

She was admitted to the intensive care unit (ICU), where she persisted with hemodynamic instability, requiring escalation of norepinephrine up to 0.93 µg/kg/min. Abdominopelvic computed tomography (CT) scan revealed diffuse colonic wall thickening consistent with pancolitis (Figure 1). Endoscopic evaluation was deferred due to hemodynamic instability and severe neutropenia, with the diagnosis of immune-mediated colitis supported by clinical presentation, imaging findings, and exclusion of infectious causes. Serum cortisol was markedly decreased (1.9 µg/dL).

Following multidisciplinary discussion with oncology and endocrinology, the final diagnosis of pembrolizumab-induced multiorgan immune-related toxicity was established, manifesting as acute adrenal insufficiency with shock (grade 4), colitis (grade 3), pancytopenia (grade 3), and thyroiditis evolving to significant hypothyroidism (grade 2). The full infectious workup (blood cultures, urine culture, stool bacterial and parasitic studies, *Clostridium difficile *assay, and viral gastrointestinal polymerase chain reaction panel was negative; thus, antibiotic therapy was discontinued.

The patient received pulse methylprednisolone 500 mg/day for three days, followed by oral prednisone 1 mg/kg/day with gradual taper over six weeks, filgrastim 5 µg/kg/day until normalization of absolute neutrophil count, and levothyroxine dose adjustment to 75 µg/day.

Clinical evolution was favorable, with resolution of fever, progressive hemodynamic stabilization, and discontinuation of vasopressor support by day 7 of corticosteroid therapy. Abdominal pain completely resolved, oral feeding (low-residue neutropenic diet) was resumed, and bowel transit normalized.

Laboratory results demonstrated hematologic recovery on day 2 of filgrastim, normalization of renal function, and a decline in C-reactive protein. Hormonal reassessment showed gradual improvement in cortisol levels and a low ACTH level that remained within the reference range. The laboratory reassessment is shown in Table 2.

**Table 2:** Laboratory obtained on day 2 after initiation of methylprednisolone and filgrastim therapy ACTH: adrenocorticotropic hormone

Test	Result	Normal range
Hemoglobin (g/dL)	7.5	12.0-13.0
Leukocytes (x10⁹/L)	8.4	4.0-10.0
Neutrophils (x10⁹/L)	1.48	1.5-8.0
Platelets (x10⁹/L)	206	150-400
Creatinine (mg/dL)	0.95	0.5-0.9
C-reactive protein (mg/dL)	0.29	<0.5
Cortisol (µg/dL)	2.6	6.2-19.4
ACTH (pg/mL)	10.2	7.2-63.6

On hospital day 9, she was transferred to the internal medicine ward in stable clinical condition, and on day 11, she was discharged home under a gradual prednisone taper, with a permanent contraindication to pembrolizumab, and short-term follow-up in oncology and endocrinology clinics for reassessment of adrenal and thyroid function.

At early follow-up, the patient remained clinically stable under adequate glucocorticoid replacement therapy, with persistent biochemical adrenal insufficiency but no ongoing hemodynamic instability or life-threatening features. Thyroid function was managed with levothyroxine dose adjustment, with outpatient endocrinology follow-up. Pembrolizumab was permanently discontinued, and immunotherapy rechallenge was not considered due to the severity of the irAEs. No recurrence of gastrointestinal symptoms or hematologic toxicity was observed during early follow-up. A timeline summarizing disease progression and development is presented in Table 3.

**Table 3:** Clinical timeline of chemotherapy, symptom onset, diagnostic findings, treatment, and recovery AKIN: Acute Kidney Injury Network; CRP: C-reactive protein; CT: computed tomography; ICU: intensive care unit; TSH: thyroid-stimulating hormone

Timeline	Key events
Baseline status	Early-stage triple-negative breast cancer (stage IA); Hashimoto’s thyroiditis on levothyroxine 25 µg/day
Neoadjuvant therapy	Initiation of carboplatin, paclitaxel, and pembrolizumab
After the 3rd pembrolizumab cycle	Onset of abdominal pain, nausea, vomiting, and profuse watery diarrhea (more than 10 episodes/12 hours)
Day 0 (admission)	Fever, hypotension, tachycardia, hypoglycemia. Laboratory evaluation: pancytopenia, acute kidney injury (AKIN II), hyponatremia, elevated CRP, mildly elevated procalcitonin, elevated TSH and free T4. Rapid progression to shock (fluid resuscitation and initiation of norepinephrine) with ICU admission
Day 1	CT showed pancolitis (no perforation or obstruction). Serum cortisol 1.9 µg/dL. Diagnosis of multisystem irAEs established
Days 1-3	Intravenous methylprednisolone 500 mg/day and filgrastim. Levothyroxine increased to 75 µg/day. Antibiotics discontinued after negative infectious workup
Day 2	Beginning of hematologic recovery
Day 4	Transition to oral prednisone 1 mg/kg/day
Day 7	Vasopressors discontinued; hemodynamic stability achieved
Day 9	Transfer from ICU to the internal medicine ward
Day 11	Hospital discharge. Pembrolizumab was permanently discontinued
Early follow-up	Clinical stability with ongoing glucocorticoid replacement for adrenal insufficiency and levothyroxine adjustment for thyroid dysfunction; no recurrence of colitis or cytopenias

## Discussion

ICIs represent one of the most significant advances in modern oncology, markedly improving survival across several malignancies [1-3]. By blocking inhibitory pathways such as PD-1/PD-L1 and CTLA-4, they restore antitumor T-cell activity but also disrupt immune tolerance, enabling autoreactive T-cell activation directed against healthy tissues and giving rise to immune-related adverse events (irAEs) [1,3,4]. Although irAEs are relatively common (15%-30%), severe or multisystem reactions remain uncommon, occurring in fewer than 5% of patients, and are associated with substantial morbidity and mortality [2,5,7]. Because their manifestations often mimic sepsis or chemotherapy-induced toxicity, a high index of suspicion and multidisciplinary assessment are crucial for early recognition. Although no single validated tool exists to reliably distinguish chemotherapy-related toxicity from irAEs or infection, a structured approach incorporating treatment timing, laboratory patterns, inflammatory biomarkers (such as procalcitonin), targeted endocrine evaluation, and response to empiric therapy versus corticosteroids can assist in differential diagnosis [2,4,5].

Diagnostic reasoning and differential diagnosis 

The patient developed shock, pancytopenia, colitis, and endocrine dysfunction after the third pembrolizumab infusion. Several features supported a diagnosis of multiorgan irAEs rather than infection or chemotherapy toxicity. The temporal pattern, toxicity appearing after multiple pembrolizumab cycles, matched the typical timing of irAEs, usually emerging after the second to fourth dose when T-cell activation peaks [3,12]. In contrast, chemotherapy-induced cytopenias or mucositis occurred within 7-14 days of drug exposure, making this pattern inconsistent with cytotoxic injury. An extensive infectious evaluation was negative: blood, urine, and stool cultures were sterile;* Clostridioides difficile* testing was negative; and notably, procalcitonin levels were only mildly elevated, a finding that is atypical for severe bacterial sepsis and further supported a noninfectious inflammatory etiology. The lack of response to empirical antibiotics further supported an immune-mediated process [2,4]. The endocrine profile revealed severe hypocortisolemia (1.9 µg/dL) with inappropriately low ACTH, confirming adrenal insufficiency. In septic shock, cortisol levels are typically elevated due to stress-induced hypothalamic-pituitary-adrenal activation; low cortisol with low or normal ACTH is characteristic of immune-mediated adrenal crisis [8,10]. Importantly, thyroid function tests showing markedly elevated TSH with concomitantly increased free T4 were obtained at hospital admission, prior to corticosteroid therapy or levothyroxine dose adjustment. This discordant pattern does not reflect simple primary hypothyroidism but is consistent with immune-related thyroid dysfunction induced by pembrolizumab, particularly in the context of pre-existing Hashimoto’s thyroiditis and acute critical illness. ICI-associated thyroiditis may present with transient or mixed-phase biochemical abnormalities, including delayed TSH normalization despite elevated circulating thyroid hormone levels, explaining the laboratory findings in Table 1 and supporting classification as grade 2 immune-related thyroid dysfunction. The pancytopenia was most consistent with immune-mediated bone marrow suppression. Alternative etiologies were considered and systematically excluded: the timing of cytopenias was inconsistent with chemotherapy-induced myelotoxicity, infectious workup was negative, procalcitonin levels were only mildly elevated, and there was no evidence of consumptive coagulopathy or hemophagocytic syndrome. Importantly, rapid hematologic recovery following corticosteroid therapy and filgrastim strongly supported an immune-mediated mechanism rather than sepsis-related or drug-induced marrow toxicity [6]. Collectively, the absence of infection, temporal association with pembrolizumab, and prompt steroid responsiveness confirmed a multisystem irAEs.

Distinguishing adrenal crisis from septic shock

Differentiating adrenal crisis from septic shock is critical, as management strategies differ markedly [8,9]. In this case, the biochemical profile of markedly low serum cortisol with inappropriately normal ACTH, associated hyponatremia, hypoglycemia, and hemodynamic instability, unresponsive to fluids and vasopressors but rapidly responsive to corticosteroids, supported the diagnosis of secondary adrenal insufficiency, most likely related to ICI-induced hypophysitis [8-10]. The absence of hyperkalemia made primary adrenal insufficiency less likely [8,9]. In accordance with American Society of Clinical Oncology (ASCO) and European Society for Medical Oncology (ESMO) guidelines, immediate corticosteroid therapy was prioritized in the acute setting, and further dynamic testing or pituitary magnetic resonance imaging was deferred [8,10]. Although rare, occurring in fewer than 1% of ICI-treated patients, immune-related adrenal insufficiency can be life-threatening if not promptly recognized [8,9].

Spectrum, rarity of multisystem irAEs, and mechanistic insights

Multisystem irAEs involving three or more organs are rare, occurring in less than 1% of patients, even with combined checkpoint inhibitor therapy [2,7]. The coexistence of adrenal crisis, pancytopenia, colitis, and thyroid dysfunction in this patient has only been sporadically reported and expands the recognized spectrum of pembrolizumab-induced multiorgan toxicity [7]. Compared with previously published pembrolizumab-related multiorgan toxicity cases, this presentation is notable for the simultaneous involvement of endocrine, including life-threatening adrenal crisis with shock at presentation (grade 4) and thyroid dysfunction (grade 2), gastrointestinal (grade 3 colitis), and hematologic systems (grade 3 pancytopenia), occurring in the neoadjuvant treatment setting for early-stage triple-negative breast cancer. The underlying mechanism reflects a breakdown of immune tolerance. PD-1 blockade promotes sustained T-cell activation and cytokine release, which can target normal tissues sharing epitopes with tumor antigens, such as the adrenal cortex, thyroid gland, gastrointestinal mucosa, and bone marrow, leading to cross-reactive immune attack [1,3,9]. Pre-existing autoimmune conditions further heighten susceptibility, as autoreactive lymphocytes and autoantibodies are already primed [8,10,13]. Patients with baseline autoimmune disease are more likely to experience irAEs and to discontinue immunotherapy due to toxicity [13]. Endocrine dysfunctions are among the most frequent and often irreversible complications of ICIs [8-10]. Adrenal insufficiency may result from autoimmune adrenalitis or secondary hypophysitis, the latter consistent with this case, given low cortisol and normal ACTH, and carries a high risk of hemodynamic collapse if untreated [8,9]. Thyroid dysfunction is also common and may occur de novo or as an exacerbation of pre-existing autoimmunity. In this patient, a flare of Hashimoto’s thyroiditis during pembrolizumab therapy supports the hypothesis that circulating antithyroid antibodies can amplify immune activation [8-10]. Routine monitoring of adrenal and thyroid function is therefore essential in all patients receiving ICIs, particularly those with pre-existing autoimmune disease [10,11]. Immune-mediated colitis, one of the most frequent gastrointestinal irAEs, can lead to hypovolemic or distributive shock if not promptly treated [3,5,7]. Early administration of high-dose intravenous methylprednisolone followed by tapering resulted in rapid clinical recovery in this case, emphasizing the importance of early intervention [11]. Hematologic irAEs such as pancytopenia are far less common but carry high mortality; they result from autoimmune destruction of hematopoietic progenitors and cytokine-driven marrow suppression. Filgrastim facilitated neutrophil recovery and allowed safe steroid tapering [6]. Recognition of this multisystemic pattern is essential, as prompt immunosuppression can be lifesaving.

Therapeutic management and guideline alignment

The management of severe irAEs follows ASCO and ESMO recommendations [11,12]. According to the Common Terminology Criteria for Adverse Events (CTCAE v5.0), the presence of adrenal crisis with shock (grade 4), grade 3 colitis, and grade 3 pancytopenia classified this presentation as severe immune-related toxicity, warranting immediate ICI discontinuation and initiation of high-dose systemic corticosteroids. Although classic adrenal crisis is typically treated with stress-dose hydrocortisone, high-dose intravenous methylprednisolone was selected in this case to address the coexistence of life-threatening multisystem irAEs, providing both prompt glucocorticoid replacement and adequate immunosuppression for severe immune-mediated colitis, pancytopenia, and shock. Following clinical stabilization, therapy was transitioned to oral prednisone with gradual tapering over four weeks, consistent with best practice. In this patient, early corticosteroid therapy resulted in rapid reversal of shock, colitis, and cytopenias without the need for additional immunosuppressive agents (such as mycophenolate mofetil or infliximab, reserved for steroid-refractory cases). Filgrastim was used to expedite bone marrow recovery [6,11,12]. Early initiation of corticosteroids does not appear to compromise antitumor efficacy when appropriately administered [11]. Endocrine irAEs often persist despite clinical recovery; central adrenal insufficiency is frequently permanent and requires lifelong glucocorticoid, and occasionally mineralocorticoid, replacement [8,9]. Thyroid dysfunction, which occurs in up to 6-13% of cases, requires regular levothyroxine titration guided by TSH and free T4 monitoring [10]. Adrenal insufficiency should always be corrected before thyroid hormone replacement to prevent adrenal crisis, and long-term endocrinology follow-up is essential to maintain hormonal balance and detect late recurrences [8-10].

Prognosis and risk of recurrence

Late-onset or recurrent irAEs can appear months to years after discontinuation of therapy [2,3,9]. Adrenal insufficiency rarely resolves completely, necessitating lifelong follow-up. Rechallenge with ICIs after a life-threatening irAEs carries a high risk of recurrence and should be considered only when no alternative therapy exists or only after complete resolution of grade 1 toxicity, and under close multidisciplinary surveillance [11,12].

Clinical implications and lessons for practice 

This case exemplifies the paradigm shift in oncologic emergencies introduced by immunotherapy. Unlike classical complications such as neutropenic sepsis or tumor lysis syndrome, irAEs may develop insidiously with nonspecific symptoms, including fatigue, diarrhea, cytopenia, or hypotension, that are easily misattributed to infection or chemotherapy [1,4,5,12]. Any ICI-treated patient presenting with unexplained multiorgan dysfunction should prompt evaluation for immune toxicity. Optimal outcomes depend on coordinated management involving oncology, internal medicine, endocrinology, hematology, gastroenterology, and intensive care teams [2,5,7,11]. Institutions should adopt standardized algorithms for ICI-related emergencies, with predefined triggers for endocrine and hematologic testing, and education of frontline clinicians to ensure timely recognition. Early diagnosis and prompt corticosteroid therapy remain the cornerstone of treatment, capable of reversing even life-threatening multisystem irAEs.

## Conclusions

Multisystem immune-related toxicity induced by ICIs, such as the combination of adrenal crisis, colitis, pancytopenia, and thyroid dysfunction observed here, represents a complex, potentially life-threatening, and frequently underdiagnosed clinical entity. Diagnostic reasoning should emphasize temporal association with ICIs, negative infectious workup, endocrine patterns of low cortisol with low ACTH, absence of chemotherapy-timing toxicity, and rapid steroid response. Early initiation of high-dose corticosteroids remains the mainstay of therapy, as the prompt clinical and laboratory response observed in this patient confirmed an immune-mediated etiology and underscores the importance of timely immunosuppressive treatment to prevent irreversible organ dysfunction when irAEs are suspected. This case demonstrates that early recognition is a critical determinant of favorable outcomes, particularly when the initial presentation mimics severe infections or other common oncologic complications. Oncologists and intensivists should maintain a low threshold to evaluate for irAEs and adrenal insufficiency in ICI-treated patients presenting with shock and pancytopenia, even when sepsis is initially suspected. Furthermore, this case highlights the need for proactive surveillance in all patients receiving ICIs, multidisciplinary management, and the establishment of institutional protocols for the diagnosis, treatment, and follow-up of these complications, ultimately improving prognosis and reducing associated mortality of this emerging class of oncologic emergencies.

## References

[1] Keam S, Turner N, Kugeratski FG (2024). Toxicity in the era of immune checkpoint inhibitor therapy. Front Immunol.

[2] Wan G, Chen W, Khattab S (2024). Multi-organ immune-related adverse events from immune checkpoint inhibitors and their downstream implications: a retrospective multicohort study. Lancet Oncol.

[3] Yin Q, Wu L, Han L (2023). Immune-related adverse events of immune checkpoint inhibitors: a review. Front Immunol.

[4] Chhabra N, Kennedy J (2021). A review of cancer immunotherapy toxicity: immune checkpoint inhibitors. J Med Toxicol.

[5] Marin-Acevedo JA, Chirila RM, Dronca RS (2019). Immune checkpoint inhibitor toxicities. Mayo Clin Proc.

[6] Kroll MH, Rojas-Hernandez C, Yee C (2022). Hematologic complications of immune checkpoint inhibitors. Blood.

[7] Astašauskaitė S, Kupčinskaitė-Noreikienė R, Zaborienė I (2024). Multiorgan toxicity from dual checkpoint inhibitor therapy, resulting in a complete response-a case report. Medicina (Kaunas).

[8] Husebye ES, Castinetti F, Criseno S (2022). Endocrine-related adverse conditions in patients receiving immune checkpoint inhibition: an ESE clinical practice guideline. Eur J Endocrinol.

[9] Martella S, Lucas M, Porcu M (2023). Primary adrenal insufficiency induced by immune checkpoint inhibitors: biological, clinical, and radiological aspects. Semin Oncol.

[10] Paschou SA, Stefanaki K, Psaltopoulou T (2021). How we treat endocrine complications of immune checkpoint inhibitors. ESMO Open.

[11] Brahmer JR, Lacchetti C, Schneider B (2018). Management of immune-related adverse events in patients treated with immune checkpoint inhibitor therapy: American Society of Clinical Oncology Clinical Practice Guideline. J Clin Oncol.

[12] Postow MA, Sidlow R, Hellmann MD (2018). Immune-related adverse events associated with immune checkpoint blockade. N Engl J Med.

[13] Eun Y, Kim IY, Sun JM (2019). Risk factors for immune-related adverse events associated with anti-PD-1 pembrolizumab. Sci Rep.

